# Tn-Seq Explorer: A Tool for Analysis of High-Throughput Sequencing Data of Transposon Mutant Libraries

**DOI:** 10.1371/journal.pone.0126070

**Published:** 2015-05-04

**Authors:** Sina Solaimanpour, Felipe Sarmiento, Jan Mrázek

**Affiliations:** 1 Department of Computer Science, University of Georgia, Athens, GA 30602, United States of America; 2 Swissaustral USA, 111 Riverbend Rd., Athens, GA 30602, United States of America; 3 Department of Microbiology and Institute of Bioinformatics, University of Georgia, Athens, GA 30602, United States of America; CNRS UMR7622 & University Paris 6 Pierre-et-Marie-Curie, FRANCE

## Abstract

Tn-seq is a high throughput technique for analysis of transposon mutant libraries. Tn-seq Explorer was developed as a convenient and easy-to-use package of tools for exploration of the Tn-seq data. In a typical application, the user will have obtained a collection of sequence reads adjacent to transposon insertions in a reference genome. The reads are first aligned to the reference genome using one of the tools available for this task. Tn-seq Explorer reads the alignment and the gene annotation, and provides the user with a set of tools to investigate the data and identify possibly essential or advantageous genes as those that contain significantly low counts of transposon insertions. Emphasis is placed on providing flexibility in selecting parameters and methodology most appropriate for each particular dataset. Tn-seq Explorer is written in Java as a menu-driven, stand-alone application. It was tested on Windows, Mac OS, and Linux operating systems. The source code is distributed under the terms of GNU General Public License. The program and the source code are available for download at http://www.cmbl.uga.edu/downloads/programs/Tn_seq_Explorer/ and https://github.com/sina-cb/Tn-seqExplorer.

## Introduction

We use the term Tn-seq to refer to a range of transposon insertion sequencing techniques that use a random transposon mutant library and high-throughput sequencing to study fitness of mutant strains and/or to identify genes that are essential or advantageous for growth under a specific set of conditions. Thanks to the novel advances in deep sequencing technologies, this technique has become useful for understanding gene function and the genetics behind microbial physiology. [1,2,3,4,5,6].

Even though the rationale and the overall technique of Tn-seq is straightforward, there are different variations in the methodology to achieve the expected results [4]. In general terms, DNA is extracted from a library of transposon mutants before and after the library is grown under controlled conditions for a fixed amount of generations. DNA is sheared in a random fashion by mechanical methods and the ends are repaired. Alternatively, DNA is fragmented by restriction enzymes. In either case, small enrichment/sequencing adapters are attached to the resulting DNA fragments. Transposon-chromosome junctions are enriched by PCR, PCR-affinity purification technique (gel size selection plus biotin probe-streptavidin magnetic beads) or the Tn-seq circle method [5]. Finally, high-throughput sequencing is used to sequence either the transposon-chromosome junctions, when the fragments are sequenced by using the primer sites in the adaptors previously attached, or the chromosomal sites adjacent to the inserted transposons, when custom-made primers that bind to the end of the transposon are used. These sequence reads are aligned to a reference genome in order to determine the locations of transposon insertions. The goal of the subsequent data analysis is to establish the exact location of each transposon insertion and the number of sequence reads mapped to each insertion site, and determine the number of insertions/reads in a specific genomic region. This information is subsequently used to identify genes with a significantly low number of transposon insertions/reads detected in the mutant library as genes that are possibly essential or advantageous for growth. The data can also be utilized to study fitness by analyzing the frequency of the insertion mutants in the population over time as a direct relation of the growth rate of the mutant strain [7].

Several methods were developed for analysis of Tn-seq data [1,3,8,9,10]. Some of these methods employ a null model to estimate the expected count of transposon insertions in each gene; if the actual count is significantly lower than the expected count the gene is considered essential [9]. An alternative approach is based on separating the populations of essential and non-essential genes using as a guide the distribution of transposon insertion densities among all genes in the genome. This technique takes advantage of the observation that the distribution is generally bimodal with essential and non-essential genes constituting separate peaks [10,11]. Another approach involves comparison between two samples from cultures grown under different conditions or extracted at different time points, where one sample is used as a baseline against which the other is compared [1,5]. In addition to these techniques, we developed a sliding window approach, which avoids comparisons among genes of variable length by comparing instead insertion counts in a sliding window of fixed size [2].

Our experience in the analysis of Tn-seq data and the considerations elaborated below influenced our goals in developing Tn-seq Explorer. We aimed to design an easy-to-use interactive application that would allow users to explore their data in order to determine the most appropriate method for detection of genes that may be essential or advantageous, or for studying fitness. Tn-seq Explorer provides useful complementary tools for data analysis, as well as flexibility in selecting parameters that are most appropriate for each particular dataset.

## Methods

### Insertion density approach

The goal of the method is to generate hypotheses on the essential or advantageous nature of specific genes for growth under different conditions, in which the mutant libraries were cultured, utilizing the basic premise that mutants with insertions in essential genes would not be viable. Note that we use the term “essential” in reference to genes that are absolutely required for growth under any condition as well as genes that are conditionally-required for growth under a specific set of conditions. The term advantageous refers to genes which are not absolutely essential, but when truncated by the transposon insertions the fitness of the mutant decreases. Essential genes and advantageous genes are characterized by a low density of insertions in the Tn-seq data.

The technique utilizing insertion density on a gene-by-gene basis was applied by Langridge *et al*. [10] and subsequently adopted by other authors. Each gene is assigned a value equal to the count of reads or unique insertions mapped to this gene, divided by the length of the gene. If the insertions/reads are distributed randomly throughout the genome the density of insertions is expected to be approximately equal for all genes (apart from random variance). However, the empirical distribution of insertion densities among different genes is generally bimodal, separating non-essential genes that are not affected or only weakly affected by selection from essential or advantageous genes that feature low insertion densities due to selective elimination of the corresponding mutants. The point separating the two peaks in the distribution can be used as a cutoff value where genes with lower insertion densities are considered essential whereas genes with higher insertion densities are assigned putative non-essential status. Although this approach can be seen as problematic because it compares insertion densities among genes of varying sizes while ignoring the fact that random variance in insertion densities is higher for smaller genes, it has been successfully used in situations where the insertion densities are high, that is, for mutant libraries with a high level of saturation [10,11].

### Sliding window approach

One particular challenge we faced in the analysis of the *M*. *maripaludis* Tn-seq data [2] was a relatively low saturation of the mutant library, which made some of the previously used methods problematic. The Tn5 transposon utilized in that work inserts at random positions in the genome, with only a weak preference for specific sequences [12,13]. However, although the Tn-seq experiments produced millions of reads, these reads were mapped to tens of thousands of unique positions in the genome, suggesting that all sequence reads came from a rather limited number of unique mutants. Polyploidy of the studied organism, *M*. *maripaludis*, further exacerbated some of the difficulties in the data analysis.

Additional challenges were posed by strong biases observed in the distribution of mapped sequence reads. If every unique transposon insertion (or unique mutant) had an equal chance to yield a sequence read the distribution of number of reads per unique insertion should be similar to a normal distribution, although it could be skewed to some extent by selection against insertions in essential genes and presence of insertion “hot spots” [14]. In reality, the distribution resembles a power law distribution, which is indicative of strong biases in how likely different mutants are to be detected by the high-throughput sequencing. For example, one of our mutant libraries was represented by 2,593,856 sequence reads which could be reliably mapped to the genome but all these reads were mapped to only 23,962 unique insertions (defined by a position in the genome and the orientation of the transposon)—an average of 108 reads per unique insertion. In one extreme, this library included nine insertions represented by >5000 reads and 256 insertions represented by >1000 reads, whereas 5679 (24%) unique insertions were represented by a single read and 7843 (33%) by 3 or fewer reads. We considered a scenario where positive selection could lead to proliferation of some specific mutants that outcompete other mutants and the wild-type strain in the culture but the genes that contained the insertions represented by high numbers of reads also contained other insertions that were represented by normal or low numbers of reads, arguing that this scenario cannot fully explain the observed data. We believe that this bias in distribution of reads per unique insertion could be an artifact of the experimental procedures in the preparation of the samples for sequencing, which involves amplification and enrichment for transposon-chromosome junctions, or it could arise from other processes that we do not fully understand.

The low level of mutant saturation also affects statistical approaches utilizing a formal null model to estimate the expected number of sequence reads mapped to a gene [3,9]. The drawback of counting all reads mapped to a gene is that sequence reads are not independent observations for the purpose of statistical evaluations, which could lead to type I errors (false positive classifications of genes as essential when rejection of the null hypothesis is in reality not justified). To avoid this issue, the counts of unique insertions per gene can be considered instead of counts of all sequence reads, while disregarding the information about the number of reads per insertion [2,10,11]. The advantage of this approach is that unique insertions are more likely to represent independent observations but the disadvantage is that counts of unique insertions per gene can be low. This in turn makes the approach based on comparing insertion densities among different genes problematic because the low counts of unique insertions exacerbate the effect of differences in gene length. To bypass the issues associated with comparing genes of different sizes, we designed a sliding window approach, which compares insertion counts in segments of a fixed length.

Our approach is conceptually similar to that used by Zhang *et al*. [8], instead of counting insertions in genes, we count insertions in overlapping windows of a fixed size. When the window size is sufficiently large (depending on the overall density of insertions in the genome) the distribution of the insertion counts is bimodal with low values corresponding to window locations overlapping with putative essential genes or possibly other essential genomic segments [2].

**Fig 1:**
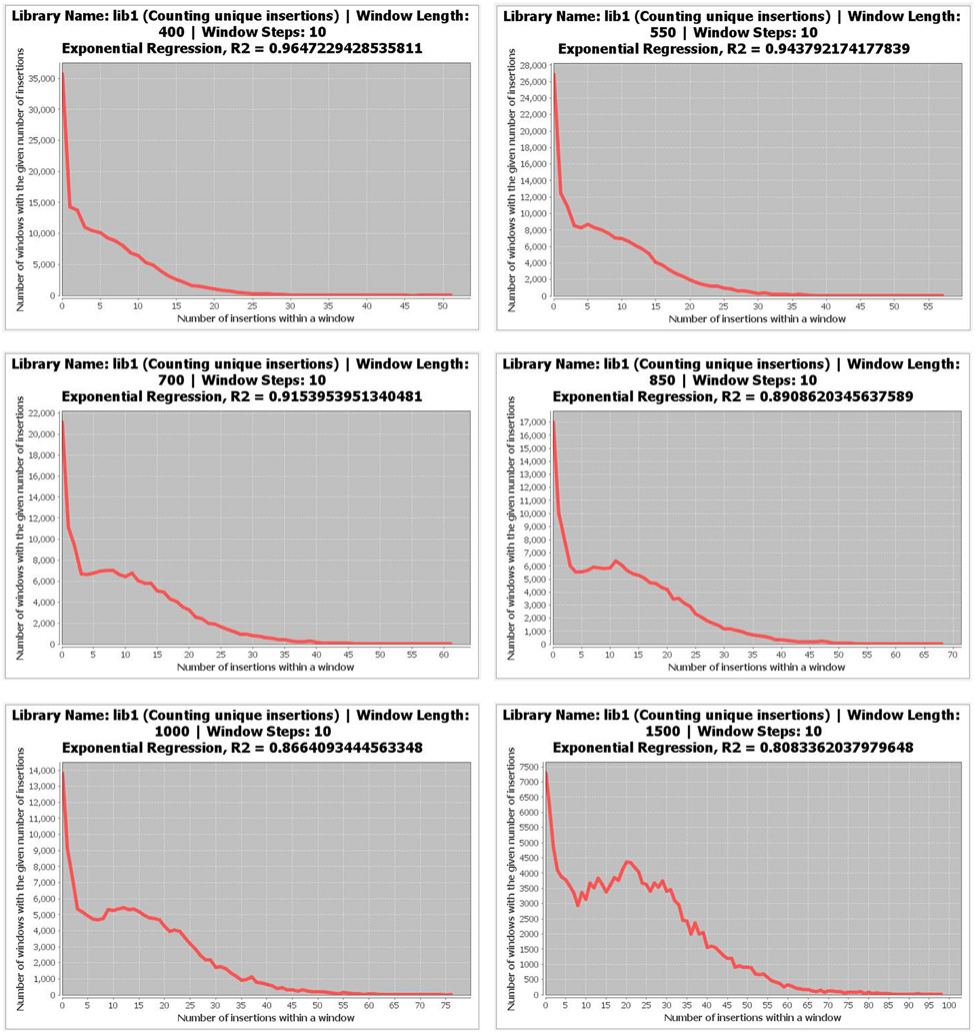
Screenshots of distribution of unique insertions per window for window sizes 400, 550, 700, 850, 1000, and 1500 bp. The analyzed genome (*M*. *maripaludis* S2) was scanned with a sliding window of the given size shifted by 10 bp in each step, counting the number of unique transposon insertions within each window. The vertical axis shows the number of window positions that yield the insertion count shown by the horizontal axis.

Tn-seq Explorer includes a tool to determine automatically an appropriate window size. This tool is based on an empirical observation that for small window sizes the distribution of insertion counts per window resembles an exponentially decreasing function and as the window size increases the distribution becomes bimodal, with the left peak corresponding to the population of essential (or advantageous) genes and the wide right peak to non-essential genes (Fig 1). Starting with a small window size, the distribution of insertion counts per window is determined and a fit to exponential function is evaluated by the coefficient of determination (R^2^). The smallest window size with R^2^ below a cutoff value is deemed appropriate for the library at hand. The cutoff value is dependent on the window size and has been determined by a heuristic analysis of empirical data and simulations. The users can override the recommended window size if they deem a different window size more appropriate for their data.

**Fig 2:**
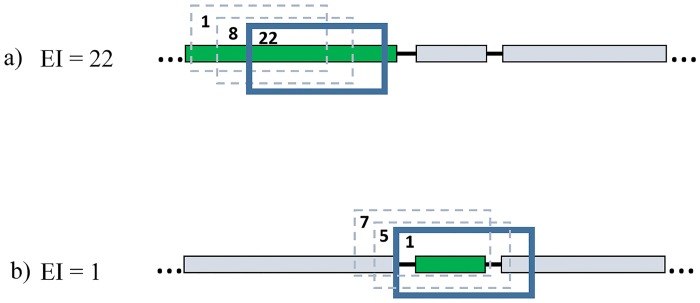
Diagram of EI assignation per gene. a) For a gene larger than the window size, the EI correspond to the window with the larger number of unique insertions among all windows embedded on that gene. b) For genes smaller than the window size, EI is the smallest insertion count among all windows that fully encompass the specific gene. 20% of the 3′ end and 5% of the 5′ end of every gene is truncated. Green rectangles represent the target gene, gray rectangles represent surrounding genes. Blue boxes represent the selected sliding window for determining the EI of the target gene; dashed blue boxes represent other sliding windows for the target gene. Numbers in the corner of the boxes represent the number of reads per window.

Once the appropriate window size is determined and counts of insertions per window are known, each annotated gene is assigned an essentiality index (EI; Fig 2). For a gene that is larger than the window size, the essentiality index is the largest insertion count in any of the windows fully embedded in that gene. For genes smaller than the window size, EI is the smallest insertion count among all windows that fully encompass the gene at hand. Note that the sliding window approach does not completely remove the uncertainty affecting small genes because if a small essential gene is surrounded by non-essential DNA all windows overlapping that gene can have high insertion counts, and as a result this gene could be incorrectly classified as non-essential (as opposed to the insertion density approach, which is more likely to classify short genes incorrectly as essential). However, the sliding window can detect clusters of essential short genes (e.g., ribosomal protein gene operons which are present in most prokaryotic genomes [2,15]).

In addition to assigning EI values to individual genes, Tn-seq Explorer also provides a list of genomic regions predicted to be essential as contiguous segments consisting of overlapping windows with low insertion counts. See ref. [2] and the related supplementary information at (http://www.pnas.org/content/suppl/2013/03/01/1220225110.DCSupplemental/pnas.201220225SI.pdf) for detailed description of the method and additional justification, as well as examples of its application.

### Additional tools for data analysis

Tn-seq Explorer provides several tools for analysis of biases in the Tn-seq data. Plots of distribution of sequence read counts per unique insertion are available to analyze biases possibly arising from mutations hot spots or artifacts in the enrichment and amplification procedures, and to decide whether analysis of read counts or unique insertion counts is more appropriate for the given mutant library. Inspecting the distribution of insertion or read counts per window of a fixed size can also reveal possible anomalies in the distribution of transposon insertions in the genome. In addition, a complete list of all unique insertions characterized by the location in the genome and orientation (strand), and the number of reads mapped to this insertion is stored as a tab-delimited table and available if a detailed investigation of particular genomic regions is needed.

Additional tools are provided for comparison between different mutant libraries. Identifying genes with large differences in essentiality indices or insertion densities in mutant libraries grown under different conditions can quickly isolate candidate genes that are essential under one set of conditions but not the other. This can be done from a tabular output in a spreadsheet format or in an interactive graph generated by Tn-seq Explorer.

Tn-seq Explorer allows for automatic adjustments of gene starts and ends because transposon insertions near the 3′ end of the gene are less likely to disrupt the gene function and therefore insertions may be found near the gene 3′ ends even in essential genes [16]. Adjustment at the 5′ end can be made to account for possibly mis-annotated translation start sites. The automatic gene start and end adjustments can be set in terms of a number of nucleotides to be excluded, as percentage of the gene size, or a combination of both. For example, the users can choose to exclude insertions located in the first 30 nucleotides and the last 20% of the gene. The default parameters are set to exclude the 20% of the gene size at the 3′ end and 5% at the 5′ end. Moreover, the genome annotation can be manually modified, for example, to add genes that may have been recently discovered but are not included in the annotations downloaded from online databases, or correct gene start and end coordinates if they are believed to be inaccurate.

## Results and Discussion

### Tn-seq Explorer features and implementation

#### System requirements

Tn-seq Explorer is a standalone Java application. It was tested on Windows, Mac OS, and Linux systems and requires Java Virtual Machine version 7 or latter (http://www.java.com/en/download/). The Bowtie2 feature in Tn-seq Explorer has been tested on Mac OS, Linux, and 64-bit Windows systems but it is not available on 32-bit versions of the Windows operating system.

#### Input and output

The primary input is the alignment of sequence reads to the reference genome in the SAM format [17]. Popular tools for alignment of sequence reads to a reference genome sequence, such as Burrows-Wheeler Aligner (BWA) [18] or Bowtie [19], can generate the SAM-formatted output. Bowtie2 [20] can be started directly from the Tn-seq Explorer menu and Tn-seq Explorer provides a brief guidance how to obtain the data using BWA. When Tn-seq Explorer runs on Linux it can attempt to download, install and run the BWA directly from the Tn-seq Explorer menu.

The gene annotation (including gene locations and functional descriptions) is another required input. For publicly available completely sequenced genomes, Tn-seq Explorer can automatically download the required files from the NCBI FTP server (ftp://ftp.ncbi.nih.gov/genomes/Bacteria/). Alternatively, the users can prepare the annotation files themselves in an appropriate format.

The primary output is a tab-delimited text file that can be opened as an Excel spreadsheet. The lines in the table relate to annotated genes and most data analysis tasks performed by Tn-seq Explorer add new columns to the spreadsheet, which contain the results of that task, such as assigning each gene an essentiality index or insertion density. Some tasks generate a graphical output or a separate file.

#### A typical session with Tn-seq Explorer

A typical session starts with the user setting up a new project by providing the information about the studied genome including the gene annotation. The next step consists of preparing the mutant library files. For the purposes of Tn-seq Explorer, each library is represented by a SAM-formatted file that contains sequence reads from a Tn-seq experiment aligned to the reference genome. The subsequent data analysis assumes that the 5′ ends of the aligned reads correspond to the sites of transposon insertions. A project can include multiple libraries, e.g., growth under different conditions, different time points, or duplicate experiments. Tn-seq Explorer processes each SAM file to identify unique insertions (characterized by a unique position and orientation in the genome) and the number of sequence reads representing each unique insertion. Most subsequent analyses provide options to count all reads or only unique insertions. At this stage, the users can investigate biases in the distribution of reads to determine the most appropriate method for data analysis. There is also an option to exclude all unique insertions represented by a single read (or a small number of reads) if the user believes that these could be false observations arising from misaligned sequence reads.

After the initial processing of the SAM file, the following step involves determining the appropriate window size if the user decides to use the sliding window method. An appropriate window size is such that exhibits bimodal distribution of insertion counts per window with separate peaks corresponding to windows with low number of insertions (presumably overlapping with essential genes or other essential segments of the genome) and windows with high number of insertions, which are unaffected or weakly affected by selection under the given growth conditions. Tn-seq Explorer includes a feature to recommend the appropriate window size automatically by evaluating the distribution of insertion counts per window but users can also visually inspect the insertion count distributions for different window sizes and override the default values.

Once the libraries are set up and the appropriate window size determined, the subsequent data analysis is performed in the menu tab ‘Manage data tables’. The primary tool in this part assigns essentiality indices to the annotated genes. Alternatively, the insertion densities (or raw insertion/read counts) can be assigned to each gene and added to the spreadsheet. To accommodate analysis of Tn-seq data obtained with Mariner transposon, which inserts specifically at TA sites, Tn-seq Explorer includes an option to normalize the insertion counts relative to the number of TA sites in a gene instead of gene size. All tools can be applied repeatedly for different mutant libraries and/or for different parameters (e.g., different adjustments of gene ends or different window sizes in the sliding window approach), and each time a new column is added to the spreadsheet. The users can edit the spreadsheet to remove unwanted data or sort or otherwise rearrange the data as long as the overall format of the table is maintained. Additional tools allow comparing data in a pair of columns, which can be helpful in identifying genes with divergent insertion densities in different libraries (e.g., different growth conditions).

#### Project file structure

Each project is assigned a folder in which the project files are stored. The files created by Tn-seq Explorer are stored as tab-delimited text files and can be modified by the user. For example, the users can modify the annotation by adding or removing genes.

#### Source code availability

Tn-seq Explorer source codes are available under the terms of GNU General Public License (http://www.gnu.org/licenses/gpl.html). We encourage users with appropriate skills to add new functionality to Tn-seq Explorer. In particular, users can adopt the Tn-seq Explorer framework and add new tabs to the menus that provide new functions, including their own methods for Tn-seq data analysis.

### Testing of Tn-seq Explorer on previously published data and comparison with alternative software for Tn-seq data analysis

#### Reanalysis of *M. maripaludis* Tn-seq data

We compared the performance of the sliding window approach implemented in Tn-seq Explorer with the online server ESSENTIALS [9] by analyzing the same data by the two programs. We used the library of *M*. *maripaludis* S2 mutants from our previously published work [2]. This mutant library was grown for 14 generations in rich medium without antibiotic selection. Sequence reads from the Tn-seq experiment were mapped to the *M*. *maripaludis* genome using the Burrows-Wheeler Aligner [18] and the resulting SAM file was used as input for both Tn-seq Explorer and ESSENTIALS. The window size in Tn-seq Explorer was set to 550 nucleotides, the value determined as optimal by the software, and ESSENTIALS was run with the default parameters. ESSENTIALS assigns each gene the value LogFC, or log-fold-change, which is a logarithm of the ratio of the observed and expected counts of sequence reads mapped to that gene. We used the LogFC values from ESSENTIALS and compared them to the essentiality indices (EI) from Tn-seq Explorer. ESSENTIALS also assigns p-values to each gene but we found the p-values to be unrealistically low and unsuitable for classification of genes as essential or nonessential. Specifically, of the 1772 genes in our data set, only 193 had the FDR-adjusted p-value >10^–5^ and 1129 genes had p<10^–50^, which suggests that the null model used by ESSENTIALS is not a sufficiently accurate representation of the studied system.

**Fig 3:**
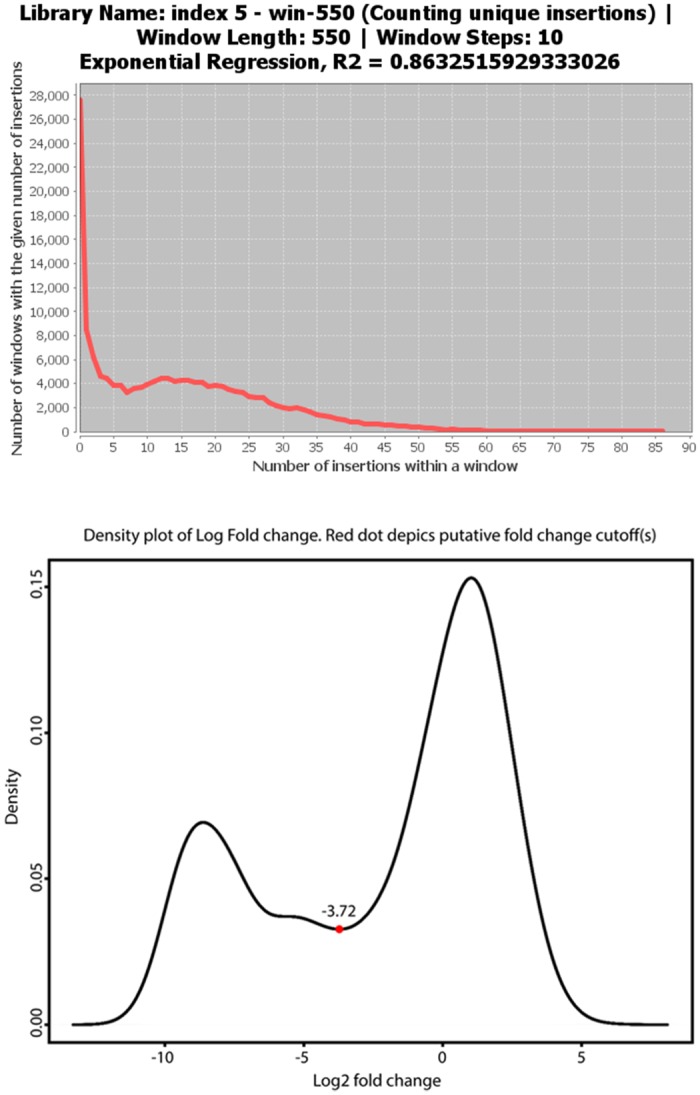
Distribution of counts of unique insertions in a sliding 550 bp window from Tn-seq Explorer (top) and distribution of LogFC values from the ESSENTIALS output.

**Fig 4:**
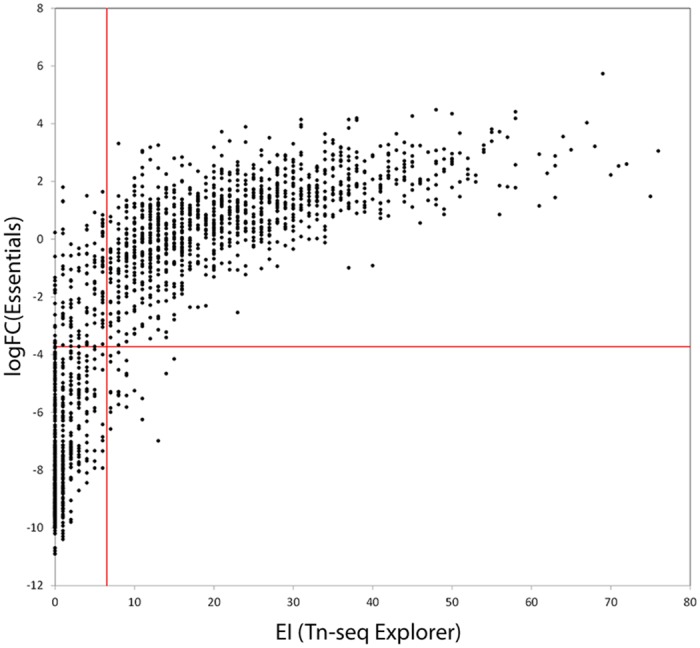
Comparison of EI values from Tn-seq Explorer and LogFC values from ESSENTIALS for the 1772 annotated genes in the *M*. *maripaludis* S2 genome.

Both LogFC and EI values show bimodal distributions with the left peak corresponding to putative essential genes (Fig 3). For EI, the minimum separating the two peaks is at 7, whereas for LogFC the minimum is at -3.72. We therefore consider genes with EI≤6 to be predicted essential by Tn-seq Explorer and those with LogFC<-3.72 predicted essential by ESSENTIALS. Using these criteria, 554 of the 1772 annotated genes are predicted to be essential by both programs, 134 are predicted essential by Tn-seq Explorer but not ESSENTIALS, 25 are predicted essential by ESSENTIALS but not Tn-seq Explorer, and 1084 are predicted non-essential by both programs. Therefore, the predictions are in agreement for 92% of genes. The agreement improves to 94% if a more conservative cutoff EI≤4 is used for Tn-seq Explorer. Fig 4 shows the comparison between the EI and LogFC values for all genes. The complete data are provided in S1 Dataset.

We compiled a list of 104 genes believed to be essential and 89 genes believed to be non-essential in the rich medium in our previous work [2]. This classification was based on considerations of the gene functions, known aspects of the *M*. *maripaludis* physiology, mutagenesis studies, and additional available information. All 104 genes believed to be essential were classified as such by Tn-seq Explorer; eighty-two had EI = 0, seventeen EI = 1, four EI = 2, one EI = 3, and none EI>3 (S1 Dataset). Five of these genes were classified as non-essential by ESSENTIALS (LogFC>-3.72), including tetrahydromethanopterin S-methyltransferase subunit G (MMP1566), DNA-directed RNA polymerase subunits N and A” (MMP1326 and MMP1364), 50S ribosomal protein L18P (MMP1418), and DNA topoisomerase VI subunit A (MMP1437). Among the 89 genes believed to be non-essential, one gene, coenzyme F420-reducing hydrogenase subunit delta, was assigned EI = 5, which is higher than any of the EI values for genes believed to be essential but below the cutoff we used, we therefore consider it a false positive prediction by Tn-seq Explorer. None of these genes were classified as essential by ESSENTIALS (see S2 Dataset for complete data). If we accept this small dataset as an accurate ‘gold standard’, we could conclude that both programs deliver a very good accuracy. Tn-seq Explorer provides slightly better sensitivity (100% as opposed to 95% for ESSENTIALS), whereas ESSENTIALS has a slightly better specificity due to one false positive from Tn-seq Explorer (100% versus 99%).

In the absence of a larger and more reliable “gold standard” in terms of an independently verified set of essential genes, it is difficult to determine rigorously which predictions are more accurate. Tables 1 and 2 provide the lists of genes which exhibit the most dramatic discrepancies between the EI and LogFC values. The most significant differences are generally limited to small genes where the classification may be unreliable and hypothetical genes. Some of the differences between the ESSENTIALS and Tn-seq Explorer may also arise from different truncations of gene starts and ends. ESSENTIALS uses a more complex formula for truncating genes at the 3’ and 5’ ends which cannot be modified by the users. In that regard, Tn-seq Explorer allows more flexibility in specifying how large portion of the gene at each end should be excluded.

**Table 1:** Genes with EI<5 (essential according to Tn-seq Explorer) but with LogFC>-1.0 (that is, strongly non-essential according to the ESSENTIALS results)

Length (bp)	Locus Tag	Gene symbol	Description	Essentiality Index	LogFC
426	MMP1235	*moaE*	molybdopterin biosynthesis MoaE	1	1.80
210	MMP1402	*-*	hypothetical protein	4	1.50
375	MMP1207	*-*	30S ribosomal protein S6e	1	1.32
612	MMP0256	*hisH*	imidazole glycerol phosphate synthase subunit HisH	1	1.30
336	MMP0022	*-*	hypothetical protein	4	0.47
225	MMP1566	*mtrG*	tetrahydromethanopterin S-methyltransferase subunit G	1	0.34
315	MMP0347	*-*	hypothetical protein	4	0.34
369	MMP0231	*-*	cysteine-rich small domain*	4	0.32
1161	MMP1364	*rpoA2*	DNA-directed RNA polymerase subunit A''	0	0.23
549	MMP1196	*-*	hypothetical protein	1	0.20
510	MMP1597	*-*	phosphatidylglycerophosphatase A	2	-0.18
309	MMP1406	*-*	translation initiation factor Sui1	4	-0.23
555	MMP1167	*-*	flavoprotein-like protein	3	-0.32
801	MMP1285	*-*	hypothetical protein	4	-0.50
345	MMP0217	*-*	transcriptional repressor-like protein	4	-0.51
711	MMP1267	*-*	hypothetical protein	3	-0.53
807	MMP1528	*pheA*	prephenate dehydratase	1	-0.56
363	MMP0465	*-*	hypothetical protein	2	-0.58
156	MMP1706	*-*	H/ACA RNA-protein complex component Nop10p	0	-0.61
693	MMP1526	*rncS*	ribonuclease III family protein	4	-0.91
1764	MMP0650	*ilvB*	acetolactate synthase catalytic subunit	1	-0.94
1086	MMP0006	*-*	3-dehydroquinate synthase	3	-0.99

*Zinc finger-like domain pfam04071

**Table 2:** Genes with LogFC<-4.0 (likely essential according to the ESSENTIALS results) but with EI>8 (that is, probably non-essential by the Tn-seq Explorer)

Length (bp)	Locus Tag	Gene symbol	Description	Essentiality Index	LogFC
1254	MMP0707	*-*	sodium/hydrogen exchanger	15	-4.15
74	RNA_7	*tRNA-Lys2*	Lys tRNA	14	-4.66
230	RNA_45	*RNaseP*	-	13	-6.99
144	MMP0151	*rpl40e*	50S ribosomal protein L40e	11	-6.25
87	RNA_32	*tRNA-Ser3*	Ser tRNA	11	-5.52
75	RNA_15	*tRNA-Arg3*	Arg tRNA	10	-5.25
207	MMP1687	*korD*	2-oxoglutarate ferredoxin oxidoreductase subunit delta	9	-5.82
85	RNA_13	*tRNA-Ser1*	Ser tRNA	9	-5.42
591	MMP0350	*-*	hexapeptide repeat-containing transferase	9	-4.72
1185	MMP0356	*-*	group 1 glycosyl transferase	9	-4.66
267	MMP1330	*-*	hydrogenase assembly chaperone hypC/hupF	9	-4.21

#### Reanalysis of Tn-seq data for *Pseudomonas aeruginosa*

We used Tn-seq Explorer to reanalyze some of the data from a previously published study of tobramycin resistance in *P*. *aeruginosa* [5]. Like our previous work on *M*. *maripaludis*, this study utilized the Tn5 transposon but the Tn-seq experimental methodology included a DNA circularization and exonuclease treatment to enrich for chromosome-transposon junctions. Because this work aimed specifically to identify genes responsible for tobramycin resistance, the data analysis centered on a comparison between mutant libraries grown in the presence and absence of tobramycin. Using the library grown in the absence of tobramycin as a control eliminates some of the uncertainties related to possible biases in transposon insertions as long as they affect both libraries approximately equally.

We downloaded the raw read data from the European Nucleotide Archive (ENA; http://www.ebi.ac.uk/ena/data/view/SRP004542) and analyzed libraries SRR073532 (14,152,566 reads) and SRR073533 (10,699,404 reads), which correspond to growth without and with tobramycin, respectively, in trial 1 reported in the original work [5]. We mapped the reads to the reference genome using BWA. This resulted in 12,642,610 and 9,830,486 reads mapped to 97,618 and 77,281 unique insertions for the two libraries, respectively. These numbers are similar to those reported by Gallagher *et al*.; small differences are expected because different software were used to map the reads to the reference genome. Because the goal of this study was to identify genes negatively selected in the presence of tobramycin by a direct comparison between the two libraries, we used the insertion density method and counted all sequence reads mapped to each gene.

To make our results comparable to those reported in the original work, we counted reads mapped to the central 80% of the genes (excluding 10% at each end as opposed to the Tn-seq Explorer default 5% at 5’ end and 20% at 3’ end), and we excluded from the analysis genes that had fewer than three unique insertions in the library SRR073532 (growth without tobramycin). The results are shown in S2 Dataset. Gallagher *et al*. [5] identified 117 genes as being affected by tobramycin selection when using as a main criterion that the number of reads mapped to the gene dropped 2.5-fold in the tobramycin library compared to the one grown in the absence of tobramycin. That is, the selection ratio (number of reads in the library grown with tobramycin divided by number of reads without tobramycin) was <0.40. Of those 117 genes, 95 have the selection ratio <0.40 in our data, additional 10 have the ratio <0.50, and two were excluded for having less than 3 unique insertions. However, we found 369 genes with the ratio <0.40 that were not identified in the original work as affected by selection (S2 Dataset). There are several likely reasons for these discrepancies: (i) we analyzed only one of the two biological replicates available whereas the original analysis utilized both replicates; (ii) Gallagher *et al*. performed additional normalizations of the read counts that we did not include in our analysis; (iii) they excluded additional genes when “manual examination of the distribution of hits and reads within the ORF betrayed questionable evidence of negative selection, even though the strict numerical criteria were satisfied” [5]. We believe that the large number of “false positives” relative to the results in the original work is in large part due to careful manual analysis of the data performed by the authors, including manual investigation of the distribution of insertions within each candidate gene, and demonstrates potential drawbacks of reliance of automated data processing. In that regard, Tn-seq Explorer provides several tools for detailed exploration of the data, including access to intermediary data files in an easy-to-read form (tab-delimited tables readable in Excel or other spreadsheet applications).

In addition to analyzing the data with the insertion density method, which is most similar to the method used by the authors of the original work, we also analyzed the two libraries using the sliding window method and counting only unique insertions. S2 Dataset shows EI values for each gene in the two libraries (EI_tobramycin+_ and EI_tobramycin–_) and the difference ΔEI = EI_tobramycin+_—EI_tobramycin–_. Sliding window size 400 bp was used for both libraries. Note that for the purpose of calculating the difference, each EI value was capped at 10 based on the reasoning that EI≥10 indicates that the gene is probably not essential under the specific growth conditions. Using difference rather than ratio is justified because the EI values refer to insertion counts in a window of fixed size and are therefore directly comparable among different genes. In this evaluation, genes affected by negative selection in the presence of tobramycin should have negative values of ΔEI. Among the 117 gene identified in the original work, 82 had ΔEI≤-3 and additional 17 had ΔEI = -2. Most of the remaining genes with ΔEI>-2 had large EI values in both libraries, suggesting that although they may provide a selective advantage in the presence of tobramycin they are probably not essential. For comparison, 911 of all 5678 annotated genes (16%) had ΔEI≤-3 (S2 Dataset). Note that these results were obtained by counting only unique insertions, which is a major deviation from the approach we used above and the one used by Gallagher *et al*., and the larger deviation from the original results is therefore not surprising. As we argued above, counting all reads is justified in most situations where the goal is a differential analysis, that is, analysis of differences in selective constraints between two libraries. However, alternative methods can be used to gain additional insights and to flag potentially incorrectly classified genes for additional scrutiny.

#### Reanalysis of *Haemophilus influenzae* Tn-seq data

We also reanalyzed some of the previously published data for *H*. *influenzae* [1]. Unlike the studies of *M*. *maripaludis* and *P*. *aeruginosa* which utilized the Tn5 transposon, this work was done with the Mariner transposon, which inserts specifically at TA dinucleotides. The main goal of this work was to identify genes required for growth in the lung. In an analogy to the analysis of the *P*. *aeruginosa* data above, the data analysis in this work utilized comparison between a library grown in murine lung and a control library grown *in vitro*.

The original study [1] classified genes as essential (those with insertion in <5% TA sites in the control library), those with *in vitro* growth defect (insertions in <40% TA sites), non-essential (those with high read counts in both libraries), and genes required for growth in lung (high read counts in the *in vitro* control, or ‘input’, library and low read counts in the library grown in the lung, or ‘output’ library). We compared the S1 (the input library) and S3 (the output library) datasets from the original publication to perform similar classification using tools available in Tn-seq Explorer. Note that there are some differences in methodology; for example, we differentiate unique insertions in opposite orientations even if the insertion is at the same site (therefore the maximum number of unique insertions is twice the number of TA sites). We also used cutoffs inferred from bimodality of distribution of reads per TA site (read density) instead of arbitrary cutoffs of 40% or 5%. Consequently, we classified genes separately for each library as essential or non-essential with the expectation that genes classified as essential by Gawronski at al. should be classified as essential in both libraries by our method, genes originally classified as nonessential should be non-essential in both libraries, genes classified as required for growth in lung should be essential in the output library but non-essential in the input library, and those associated with growth defects in the *in vitro* library may be essential in both libraries but could also fall into other groups because this classification is based on less stringent criteria than the essential genes. Genes excluded or not classified in the original work were excluded from our analysis as well.

The read density cutoffs (number of reads divided by number of TA sites in a gene) were set to 0.58 and 0.90 for the S1 and S3 libraries, respectively. These values correspond to the minimum separating the main left and right peaks in the read density distribution obtained with Tn-seq Explorer’s default parameters. The results are provided in S3 Dataset. All genes classified as essential by Gawronski at al. are classified as essential in both libraries by Tn-seq Explorer (read densities lower than the cutoffs). Of the 900 genes classified as non-essential by Gawronski *et al*., 23 are classified as essential in the library S3 (output library) and none in S1 (input library). However, most of these 23 genes have the read densities close to the cutoff, possibly suggesting that the 0.90 cutoff we used is too high. Among the 136 genes classified as required for lung infection in the original work, we classified 12 as non-essential in both libraries, whereas 121 were classified as expected, that is, essential in library S3 but non-essential in S1. Interestingly, 3 of the genes found to be required for infection by Gawronski *et al*. are not included in the current genome annotation. All genes classified by Gawronski *et al*. as ‘Inferred in vitro and in vivo growth defects’ are classified as essential in library S3 and ~40% (37 of 98) were also essential in library S1. Most of genes described by Gawronski *et al*. as ‘Inferred in vitro growth defect’ were essential in S3 and some also in S1. The mixed results for the latter two groups are not surprising because we used only binary classification assigning each gene an essential or non-essential status whereas Gawronski *et al*. used a third class, ‘inferred growth defect’, to cover the area of uncertainty. The results can be considered in good agreement with those in the original work despite significant differences in the methodology used, use of different software in mapping the reads, and that we used only one of two input libraries.

## Conclusions

We designed the Tn-seq Explorer as an alternative to existing software to help biologists explore and analyze Tn-seq data towards understanding fitness and gene essentiality. One important observation we made in the analysis of Tn-seq data is that they can be affected by apparently non-random biases and that each dataset may require a different approach to its analysis and/or use of different values of parameters. This is why Tn-seq Explorer provides tools for interactive exploration of the Tn-seq data, alternative data analysis techniques, and flexibility in parameter settings to allow the users to determine the most appropriate approach for each specific experiment. Results obtained with tools implemented in Tn-seq Explorer are generally in good agreement with those obtained by other methods, although the comparison with the original analysis of *P*. *aeruginosa* data [5] suggests that careful manual investigation of transposon insertions within individual genes can reduce the number of false positive classifications.

## Supporting Information

S1 Dataset Comparison of Tn-seq Explorer and ESSENTIALS results for *M*. *maripaludis* mutant library grown in rich medium. (XLSX)

S2 Dataset Reanalysis of data for *P*. *aeruginosa* and comparison with results in the original work. (XLSX)

S3 Dataset Analysis of the *H*. *influenzae* data using insertion density normalized by number of TA sites and counting all reads. (XLSX)
